# Navigation and Robotics in Interventional Oncology: Current Status and Future Roadmap

**DOI:** 10.3390/diagnostics14010098

**Published:** 2023-12-31

**Authors:** Georgios Charalampopoulos, Reto Bale, Dimitrios Filippiadis, Bruno C. Odisio, Bradford Wood, Luigi Solbiati

**Affiliations:** 1 2nd Department of Radiology, University General Hospital “ATTIKON”, Medical School, National and Kapodistrian University of Athens, 1 Rimini Str, 12462 Athens, Greece; charalampopoulosg@gmail.com; 2 Interventional Oncology/Stereotaxy and Robotics, Department of Radiology, Medical University of Innsbruck, 6020 Innsbruck, Austria; reto.bale@i-med.ac.at; 3 Department of Interventional Radiology, The University of Texas MD Anderson Cancer Center, Houston, TX 77030, USA; bcodisio@mdanderson.org; 4 Interventional Radiology and Center for Interventional Oncology, NIH Clinical Center and National Cancer Institute, National Institutes of Health, Bethesda, MD 20892, USA; bwood@cc.nih.gov; 5 Department of Radiology, IRCCS Humanitas Research Hospital, Rozzano (Milano), Italy and Department of Biomedical Sciences, Humanitas University, Pieve Emanuele (Milano), 20072 Milano, Italy; lusolbia@gmail.com

**Keywords:** navigation, robotics, ablation, biopsy

## Abstract

Interventional oncology (IO) is the field of Interventional Radiology that provides minimally invasive procedures under imaging guidance for the diagnosis and treatment of malignant tumors. Sophisticated devices can be utilized to increase standardization, accuracy, outcomes, and “repeatability” in performing percutaneous Interventional Oncology techniques. These technologies can reduce variability, reduce human error, and outperform human hand-to-eye coordination and spatial relations, thus potentially normalizing an otherwise broad diversity of IO techniques, impacting simulation, training, navigation, outcomes, and performance, as well as verification of desired minimum ablation margin or other measures of successful procedures. Stereotactic navigation and robotic systems may yield specific advantages, such as the potential to reduce procedure duration and ionizing radiation exposure during the procedure and, at the same time, increase accuracy. Enhanced accuracy, in turn, is linked to improved outcomes in many clinical scenarios. The present review focuses on the current role of percutaneous navigation systems and robotics in diagnostic and therapeutic Interventional Oncology procedures. The currently available alternatives are presented, including their potential impact on clinical practice as reflected in the peer-reviewed medical literature. A review of such data may inform wiser investment of time and resources toward the most impactful IR/IO applications of robotics and navigation to both standardize and address unmet clinical needs.

## 1. Introduction

Percutaneous interventions in Interventional Oncology require precision and accuracy to maximize safety and efficacy while keeping radiation dose and procedure time as low as possible. The evolution of hardware, computer science tools, computational power, navigation, imaging, and robotic technology has improved the targeting and treatment of small and difficult-to-reach lesions with minimally invasive image-guided therapies. Navigation and robotic guidance systems have increasingly been used in IO, aiming not only to improve precision and safety but also to minimize the effects of operator skill and experience variabilities while reducing radiation exposure. These systems have been studied in multiple phases of development and in a variety of percutaneous image-guided therapy applications, including tumor biopsy and ablation, drainage, vertebroplasty, drilling of osteochondral lesions [1], percutaneous pelvic fracture fixation [2], as well as nerve and facet blocks and neurolysis [3,4,5,6]. Most systems available in the market are designed for percutaneous non-vascular applications with a spectrum of image guidance modalities, including computed tomography (CT), cone beam CT (CBCT), magnetic resonance imaging (MRI), positron emission tomography (PET), fluoroscopy, ultrasonography either independently, or a combination by the application of fusion imaging tools. Depending on the system and role of the operator, the interventional procedure can be performed in an autonomous or semiautonomous way, with the potential for remote operation. Navigation and robotic systems can be patient, ceiling, floor, or table-mounted and can interface with imaging systems as plug-in add-ons, with a spectrum from autonomous to completely integrated architecture.

Presently, percutaneous ablation is usually applied with a curative intent, aiming to provide local tumor control in a wide variety of both primary and metastatic tumors. In such cases, ablation with clear margins (AO) improves technical and clinical efficacy as well as local control and oncologic outcomes. Pre-requisites for a successful ablation procedure include proper selection of lesions and patients, as well as real-time imaging confirmation of margins, which implements and necessitates three-dimensional software technology [7,8,9,10].

The present narrative review paper focuses on the current role of percutaneous navigation systems and robotics in Interventional Oncology procedures. The currently available alternatives are reviewed, including their impact on clinical practice, as shown in published clinical experience. This is not a systematic review of the literature. We conducted the search in three databases (Pubmed/MEDLINE, Embase, and Google Scholar). The keywords (“robotics”, “navigation”, and “stereotactic”) were searched. There was no limitation date with the search conducted until May 2023. Non-English studies and case reports were excluded from this study. All references to the obtained articles were also evaluated for any additional information.

## 2. Basics of Navigation and Robotic Systems

Percutaneous image-guided needle procedures are dependent on accurate planning and precise needle placement during the procedure to ensure safety and efficacy. Sophisticated devices assist image-guided IO procedures by enabling the guidance of instruments and needles into the patient to reach target lesions, sometimes also providing real-time spatial feedback, which is a requisite to improved targeting via iterative (closed or open-loop) procedures [11]. These systems allow for the laser-, hybrid-, deep learning-, optical-, or electromagnetic-based tracking of the instruments to be used during the interventional procedure [12]. Real-time imaging and the use of image fusion additionally improve visualization of the needle track, targets, and treatment effects [3,13,14].

Laser guidance has already been used for decades to improve accuracy during CT-guided percutaneous interventions. The puncture point and trajectory angle of the needle are shown exactly by a laser ray on the patient’s skin. Laser guidance systems can be hand-held, mounted to a circular rail placed immediately in front of the CT gantry, or fixed elsewhere in the CT room or gantry itself [15,16]. Laser guidance has been used for biopsy and drainage procedures [15,17], offering improved accuracy, speed, and safety of the interventions and reducing radiation exposure during the procedure [17].

Augmented reality (AR) navigation shows comparable accuracy to CBCT-guided fluoroscopy but significantly reduces procedure time and radiation exposure [14,18]. Radiopaque fiducials are attached to the patient’s skin and on the needle to be tracked in real time. After the CT scan with fiducials is taken, the software allows for automatically segmenting the organs of interest and the target lesion(s) and automatically registering the fiducials on the patient with the ones segmented by the software. By using AR goggles, the physician can very rapidly see the superimposed 3D reconstruction of organs and lesions on the patient in the correct location with very high precision. Moreover, the fiducials placed on the needle show when the needle reaches the target lesion with high accuracy [19,20,21]. Improvements were reported for depth perception and understanding of spatial anatomic relationships [22]. The use of radiation is not required, but fluoroscopy may enable real-time monitoring and adjusting for respiratory deformation or needle deviation. Thus, AR can be used in conjunction with CBCT-guided fluoroscopy imaging to overcome this drawback [14,23].

Cone-beam CT is an imaging modality that is based on the rotational movement of a C-arm equipped with a flat panel detector around the patient, obtaining multiple radiographic images during unit rotation [24]. Acquired data are automatically reconstructed into three-dimensional CT-like datasets that can be analyzed and postprocessed on a workstation, like multidetector CT, but with a smaller field of view. Visualization of skin and deep targets may, however, be challenging in large patients. Navigational software can be combined with cone-beam CT and fusion of fluoroscopic images on the preliminary cone-beam CT, allowing for the monitoring of the trajectory tract and improving accuracy, safety, and effectiveness (even in the case of complex needle paths) without the need for additional equipment [25,26]. Cone-beam CT has been used for percutaneous tumor biopsy and ablation [27,28,29,30,31,32]. Limitations of this technique are artifacts related to rotational X-ray imaging, greater risk of motion artifacts, and limited field of view compared to CT, necessitating trained staff and careful planning before image acquisition to ensure that the appropriate region of interest is included in the field of view [11,24].

Frameless stereotactic navigation systems consist of an electromagnetic or optical localization system, a workstation, a monitor, dedicated software, and various trackable instruments, including ultrasound probes. They allow for localizing a point within the patient in the 3D coordinate system of a CT or MR image dataset in real time. In addition, the navigation system software allows for the planning of needle trajectories in a cartesian coordinate system. The frameless stereotactic navigation systems continuously calculate spatial positions, allowing for the guidance of an instrument or an ablation probe along a controlled path to the target using a virtual line [11].

A crucial part of the optical and electromagnetic navigation workflow is image-to-patient registration. It is achieved by using markers (fiducials, trackers, or references) during pre-interventional image acquisition, so, subsequently, these markers can be linked together and referenced for registration to visualize the trace [3,14,33].

**Figure 1:**
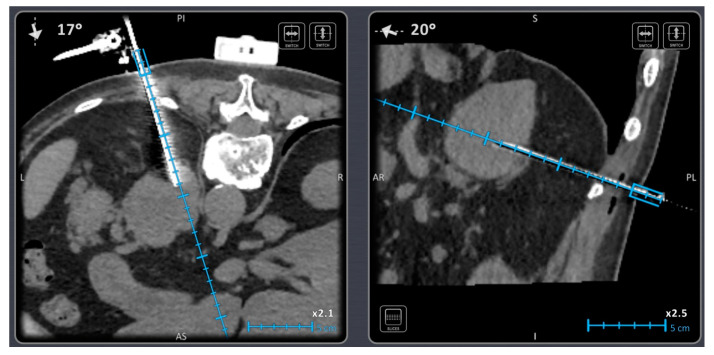
A 58-year-old male patient with a mass in the left adrenal gland. Biopsy was performed using an electromagnetic navigation device.

Electromagnetic (EM) navigation utilizes an EM field generator that is placed in proximity to or on the patient and generates a local EM field surrounding the target anatomy (Figure 1) [11]. Fiducial and EM sensors are used so they can be localized within the EM field, and their position can be calculated in relation to each other or to imaging and the patient [34]. The EM field is incompatible with MRI [11,13,35], and large metallic objects may decrease the localization accuracy.

Optical tracking utilizes video cameras or sensors detecting infrared or visible light, which is emitted or reflected by fiducials. A free line of sight between the cameras and optical markers is, however, required [36,37]. Both electromagnetic and optical navigation systems have successfully been used to define, localize, and reach lesions that are difficult to access or even inaccessible with conventional CT techniques. This has been associated with lower procedural times and radiation dose, number of needle adjustments, and skin punctures [35,38,39,40,41,42]. By combining optical navigation systems with a rigid aiming device, the accuracy of needle/probe placement can even be improved [43]. This stereotactic approach has been successfully applied in various anatomical regions, including liver [44,45,46].

**Figure 2:**
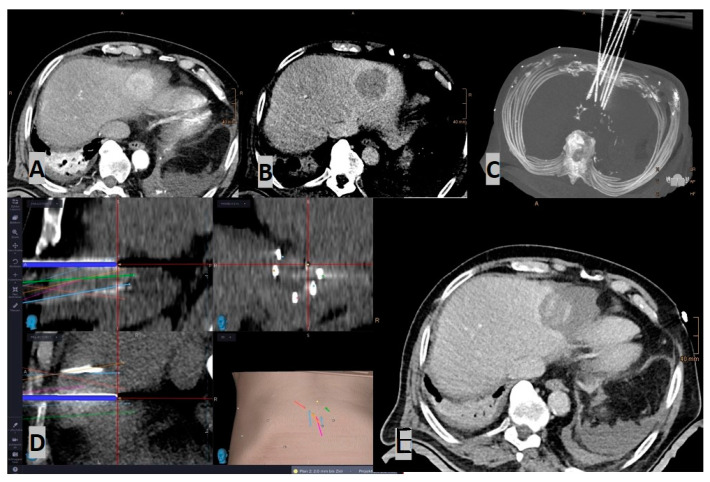
Case of a stereotactic radiofrequency ablation (SRFA) in an 85-year-old male with a sub-cardiac HCC (4.8 cm): (**A**) Arterial phase planning CT; (**B**) Portal-venous phase planning CT; (**C**) MIP of the control CT, showing in total 7 inserted coaxial needles; (**D**) Screenshot of the frameless stereotactic navigation system: Superposition of the needle control CT on the planning CT, with the pathways showing precise placement of all needles; (**E**) Contrast-enhanced control CT (portalvenous phase), showing the large ablation zone covering the HCC, including a sufficient safety margin, which was confirmed by image fusion.

**Figure 3:**
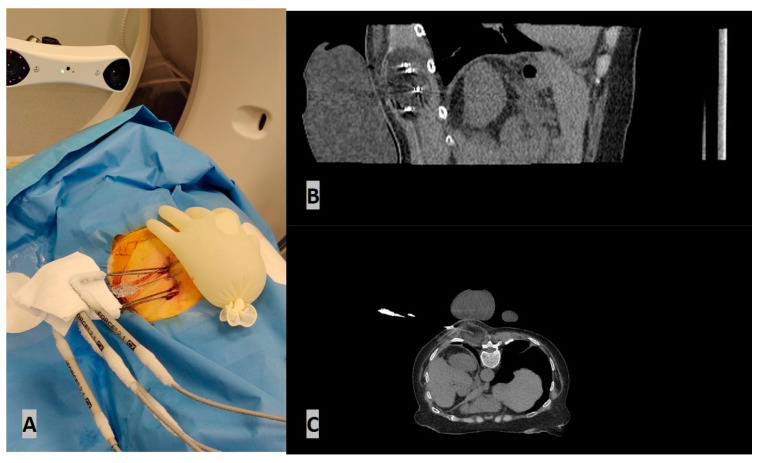
An 82-year-old female RCC patient with multiple metastatic lesions was treated with percutaneous cryoablation of a soft tissue mass in the posterior paravertebral muscles for pain palliation. (**A**) The ablation procedure was performed under CT guidance using a robotic navigation device. (**B**) Four cryoprobes were placed under CT guidance using the robotic navigation device. (**C**) Axial CT scan during ablation illustrating the ice ball and a sterile glove filled with warm water placed on the skin for protection.

While the aiming device in passive navigation systems is adjusted manually, robot-assisted systems carry out these settings (semi-)automatically Figure 2 and Figure 3.

Robotic systems provide active or passive guidance for the placement of needles and instruments. Prior to this procedure, the target is properly visualized using different image guidance modalities, such as computed tomography (CT), cone-beam CT, fluoroscopy, ultrasound, or magnetic resonance imaging (MRI) (Figure 2). Using the imaging data, a registration of the position of the robotic device versus the patient is performed, and a robotic arm moves to the proper position (often with a prior calibration confirmation). The arm is linked to a predefined entry point, angle of insertion, and depth of target (Figure 3). The correct position is confirmed by procedural imaging, and the insertion of instruments is performed through a holder mounted on the hand (“end-effector”) of the robotic arm [3,4]. The main advantage of percutaneous robot-guided interventions is the prevention of unnecessary radiation exposure while ensuring the precision of the procedure [47,48]. Some robotic systems may be used in real time with CT fluoroscopic or CBCT-registered fluoroscopy to monitor the depth and angle of insertions, accounting for respiratory motion.

## 3. Clinical Applications and the Literature Evidence

The feasibility and safety of the robotic-assisted insertion of biopsy introducer needles have been assessed with a variety of systems in a spectrum of studies, including different target locations [49,50,51,52,53,54,55,56,57,58,59,60,61]. Biopsy sessions were feasible in all patients with accurate needle targeting of the lesion [49,50,51,52,53,54,55,56,57,58,59,60,61]. The performance of a robotic system for CT-guided lung biopsy in comparison to the conventional manual technique was evaluated in a study of 100 patients, which showed that robot-assisted CT-guided lung biopsy was safe, with high diagnostic accuracy, and reduced procedure times and radiation doses in comparison to the conventional freehand technique. In this large study, the precision of needle positioning, diagnostic performance, and rate of complications were similar in patients treated with either robotic-assisted or conventional manual techniques [49]. Evaluation of an electromagnetic navigation system for lung biopsies reported that the system was safe, efficient, and reliable when compared to standard CT guidance, with high diagnostic yield and comparable or not significantly different needle insertion times and complication rates [59].

Prostate cancer is the most common malignancy in men, and the most common biopsy method was traditionally for decades freehand transrectal ultrasound, but a targeted biopsy approach guided by multiparametric MRI has become the current trend, if not standard for certain situations [50]. Robotic systems have been clinically used for transrectal [50,51], trans-gluteal [52,53], and trans-perineal [54] prostate biopsies. Schouten et al. evaluated a robotic device for transrectal MRI-guided prostate biopsy in eight patients, achieving comparable accuracy and speed in comparison to patients that underwent manual biopsy; authors reported that the robotic technique demanded higher technical effort, although it prevented the need to move the patient in and out of the scanner for manipulation and imaging of the needle [51]. Robot-assisted transrectal ultrasound-guided prostate biopsy has been successfully used in a small study including five patients and achieved the millimeter targeting accuracy required for biopsy of clinically significant prostate cancer [50]. A robotic system has been used for MR-guided trans-gluteal prostate biopsy in 20 patients, showing sufficient histopathologic assessment in 19 patients, with high accuracy in needle placement and no procedure-related complications [52]. A single-center nonrandomized prospective trial evaluated prostate-specific membrane antigen (PSMA) positron emission tomography (PET)-guided trans-gluteal prostate biopsy by using an automated robotic arm in 56 subjects [53]. This study has shown that the robotic approach was safe, with a high diagnostic yield of prostate cancer for PSMA-avid lesions, with a low rate of minor complications [53]. A mixed prospective–retrospective study evaluated the utility of a robotic needle-guidance template device (motorized template with an ability to set the needle insertion hole with a control resolution of 0.001 mm) for in-bore 3T trans-perineal MR guided prostate biopsy as compared to a manual template in a study involving 96 patients; this study has shown that the robotic needle-guidance template resulted in better targeting accuracy, more positive tissue from the cancer core, and reduced overall procedure time when compared with the manual approach [54]. Diagnostic rates were similar between the two groups, and there were no statistically significant differences in complication rates between the groups [54]. However, from an outsider’s perspective, one should mention the added cost as well as the complexities of in-gantry procedures. A study evaluating an electromagnetic navigation system for percutaneous CT-guided procedures, including biopsy and drainage accuracy, showed that it was improved by a factor of 2, and significantly lower acquisitions were performed during the utilization of the system [62]. EM tracking for prostate US/MRI fusion biopsy has become a standard method for increasing detection rates of cancer and improving the classification of cancer [63,64]. Navigation tools for prostate cancer have markedly altered the way prostate cancer is diagnosed and managed via cost-effective and accurate application of imaging information in an office setting for biopsy or focal ablation with fusion or robotic-guided laser, HIFU, IRE, or cryoablation. Robotic systems have been clinically applied to a variety of percutaneous ablative procedures, including a plethora of locations and tumor histologies [65,66,67], with excellent results [68,69]. Numerous studies upon navigation-guided or robotic arm-assisted needle positioning during thermal ablation report high rates of feasibility, significant technical ease, and ergonomics, with improved targeting accuracy, reduction in patient radiation dose, and increased procedural performance related to probe placement by a lower number of insertions and readjustments during ablation when compared to manual guidance [36,37,38,39,40]. Specifically for robots, a single insertion without adjustment was achieved in 71% of targeted lesions, whilst for EM navigation systems, accuracy was improved by a factor of 1.6, with a significantly lower number of CT acquisitions and radiation doses noted [70]. Navigation techniques and robotic assistance are particularly useful when treating technically challenging lesions, when multiple needles are required to ensure a large ablation area is achieved, and when the operator must perform the procedure at complex angles. For robotic systems that do not insert automatically (and only guide), the actual insertion of the instruments is performed by the physician, which preserves the haptic experience, allowing for the early detection of incorrect needle positioning. Therefore, utilization of navigation and robotic systems constitutes an attractive option in the management of challenging liver tumors, such as lesions hidden in US or to non-contrast-enhanced CT, large-sized tumors, or tumors located in technically difficult locations, such as in proximity to hepatic venous confluence, diaphragm, hepatic capsule, and heart, among others [71,72,73,74,75,76].

Apart from improving the efficacy, safety, and variability of ablative techniques, navigation, and robotic systems also contribute to the education of students, residents, and less experienced IOs. Navigation systems allow for the learning curves to be shortened, thus allowing the less experienced practitioners to have performance metrics like the experienced or expert [18,77]. As robotic procedures are becoming more widespread, early exposure to their applications for medical students and residents could be an integral part of training, helping them to have more meaningful observation experiences and allowing them to recognize the significance of utilizing such technologies in everyday clinical practices or for training. In a comparative trial evaluating the results of a novice with those of experienced interventional radiologists (IRs) for stereotactic radiofrequency ablation (SRFA) in terms of safety, technical success, and local tumor control, it has been shown that favorable outcomes could be achieved even by inexperienced operators with minimal supervision [77].

Navigation and Robotic systems have been clinically used for other minimally invasive percutaneous procedures, including nerve and facet block, neurolysis, peripheral bone and spine ablation, as well as vertebral augmentation and percutaneous bone fixation procedures [5,34,35,36,37,38,55,78,79,80]. In all these studies, apart from feasibility, safety, and efficacy, radiation dose reduction was also shown. Furthermore, the lack of limitation in acute angles was another advantage noted [81,82].

**Figure 4:**
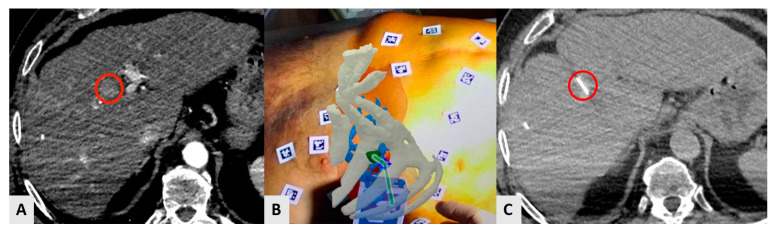
A 73-year-old male HCC patient was treated with percutaneous radiofrequency ablation in segment VIII. The ablation procedure was performed under CT guidance using an AR navigation device. (**A**) The small HCC (red circle) is visible in arterial phase. (**B**) AR guidance from the physician’s point of view. The liver is in red; the bones of thoracic case are in white; the liver vessels are in light-blue, and the lesion is in green. The green line shows the trajectory that connects the tip of the needle to the center of the target in real time. (**C**) Axial CT scan showing the tip of the needle (red circle) precisely located in the center of the lesion after the guidance by AR.

Augmented reality (AR) can also be used for imaging guidance and navigation in IO procedures using stationary, hand-held, or wearable devices (Figure 4) [14,83]. Needle trajectory is visualized on smartphones or smart glasses, providing a direct line of sight and real-time access to patient information, improving reproducibility, shortening learning curves, and reducing interobserver variability [14] of the interventional techniques [14,18]. In a study of eight patients, AR was used as a unique guidance modality for percutaneous thermal ablation of liver tumors [20]. Technical success was obtained in all cases with no complications or operator cybersickness (Figure 4). The same AR system was employed for bone biopsies in eight patients and proved to decrease radiation exposure, and the number of CT passes compared to the traditional CT guidance [21].

Globally, the need for medical imaging studies is increasing, with the number of Radiologists remaining the same. From a diagnostic point of view, AR, apart from overcoming geographical barriers, results in more accurate and efficient patient care by allowing for real-time visualization of complex medical data with exceptional precision. Regarding IR/IO techniques, the utilization of AR offers clear advantages, including, among other things, the short learning curve, the reduction in performance variability among users with different levels of experience, as well as the projection of treatment information overlay directly on the procedural environment and not in a monitor away from the patient; all these render AR significantly less expensive [77].

## 4. Where Are We Heading?

While the utilization of robotics and navigation is rapidly growing in everyday clinical practice, it is a fact that medical students and residents still have limited experience and hands-on familiarity. To enhance the engagement of medical students and residents with robotics, the inclusion of their application in the current curricula is a pre-requisite and will reduce reliance upon apprenticeships later in training or early careers. Once this is achieved, educators will be able to consider hands-on and other simulation programs for medical students and residents aiming to shift training in robotics earlier in their careers, during IR and other specialty/subspecialty training. The addition of navigation and robotics skills and simulation to the core curriculum of IR residency and training pathways might encourage earlier engagement of these paradigm-shifting technologies. As minimum ablation margin assessment and verification tools gain traction and evidence as critical elements for ablation success, tools to train will be even more critical. Utilizing navigation and robotic systems may serve as a useful learning tool in medical training, helping inexperienced operators understand their own inaccuracies and refine their skills. Thus, the end result will be related to learning curve reduction, improved technique performance, and greater patient safety.

Furthermore, the current limitation of navigation and robotic systems is the challenge of funding limitations, thus hindering widespread adoption and utilization. Vendors could start reaching out to users with more flexible and less rigid financial models before the discussion for reimbursing navigation systems and robotics in IR procedures has properly begun.

Finally, there is a need to develop a system that will contribute to ease of use, reliability, and repeatability in all the chronological steps of a procedure, starting from simulation to performance (through navigation) and verification of success. Published results suggest that we are moving toward the goal of ablation as a curative alternative to a surgical procedure. Verification of ablation zone margins by a three-dimensional software (or hardware–software platform) is key for the evolution of ablative procedures [7,8,84,85,86]. It goes without saying that navigation and robotic systems of today and tomorrow should include such verification systems.

Magnetic resonance imaging (MRI) can be used for imaging-guided procedures, providing real-time navigation along with higher resolution images and better soft tissue contrast or functional flow, dynamic, or molecular data when compared to computed tomography (CT), ultrasound, and X-ray. Experimental and clinical research in the areas of interventional tools, endovascular micro-robots, and closed-loop controlled MRI robots is trying to provide solutions for challenges such as MRI-complaint propulsion and control, navigation of medical devices through the body, clinical adaptability, ergonomics, cost, specialized equipment, and regulatory issues [87]. At present, MRI-compatible medical devices remain an emerging field, however, with immense potential clinical impact. MR-guided HIFU systems have shown promise in early trials for the brain, liver, kidney, prostate, and bone cancers. Robotic histotripsy is in early trials for certain liver cancers. Targeted navigation toward neoantigens identified within tumors partly treated with immunotherapies may also provide future pathways toward the use of robotic and navigated needles in IO.

## 5. Conclusions

Navigation or Robotic guidance offers high-accuracy needle placement and reduces the number of manipulations. Utilization of these technologies could contribute to expanding the limits and indications by treating lesions in extremely challenging locations during a technically easier, shorter, and more cost-efficient session. However, navigation systems and robotics for IO procedures are not a panacea, a solution, or a remedy for all difficulties, and they will never be a substitute for clinical judgment. They should be used as tools for providing better and more reliable clinical care for our patients. Soon, these systems will be invaluable tools assisting all operators in implementing their clinical thought and judgment in practice. Further comprehensive and integrated ergonomics that can be applied to pre-, during-, and post-procedural challenges are requisite to broader adoption. Standardization of tools, techniques, and training methods will allow the field to report meaningful results more accurately, with higher impact and value. The benefits of precision medicine are clear and will create a demand that robotic and navigation tools be used for better and more uniform outcomes in specific settings. Robotic, navigated, and stereotactic multimodality image-guided procedures are here and will only grow as we reach consensus on their absolute requirement to ensure the best outcomes. 

## Data Availability

No new data were created.
